# The Occurrence of and Distress From Pathogenic Beliefs: Examining Pathways From Childhood Adversity to Psychopathology

**DOI:** 10.1002/cpp.70163

**Published:** 2025-10-20

**Authors:** George Silberschatz, Xiaochen Luo, James McCollum, David Kealy

**Affiliations:** ^1^ Department of Psychiatry and Behavioral Sciences UCSF San Francisco California USA; ^2^ Department of Counseling Psychology Santa Clara University Santa Clara California USA; ^3^ San Francisco Psychotherapy Research Group San Francisco California USA; ^4^ Department of Psychiatry University of British Columbia Vancouver Canada

## Abstract

**Introduction:**

Pathogenic beliefs are dysfunctional beliefs that impede the pursuit of adaptive goals, which have been shown as key pathways between early childhood trauma and psychopathology. This study examined whether the occurrence of pathogenic beliefs and the distress from having pathogenic beliefs, which were assessed through the latest version of the pathogenic belief scale (PBS‐21) separately, would both mediate the childhood adversity‐outcome relationship, and whether reactivity of pathogenic beliefs (standardized difference between distress and occurrence) moderates them.

**Methods:**

A total of 390 adult participants with prior psychotherapy experience were recruited from the United Kingdom online. Participants completed self‐report measures assessing perceived adverse parenting, psychopathology (depression, anxiety, general distress and interpersonal problems) and pathogenic beliefs (occurrence, distress and reactivity).

**Results:**

Both occurrence and distress of pathogenic beliefs fully mediated the relationship between adverse parenting and depressive/anxiety symptoms and general distress, and partially mediated interpersonal problems. Reactivity significantly moderated the pathway from adverse parenting to pathogenetic beliefs. Individuals who reported more adverse parenting and were categorized as having higher reactivity were more likely to develop pathogenic beliefs and experience greater psychological distress.

**Discussion:**

These findings align with prior research showing that pathogenic beliefs are key in the association between childhood adversity and psychopathology. The study supports the validity of the PBS‐21 and demonstrates the value of assessing the occurrence of pathogenic beliefs and the distress from having these beliefs separately. Reactivity to pathogenic beliefs may serve as a clinically meaningful marker for identifying individuals with resilience and tailoring interventions accordingly.

## Summary

- The subjective meanings attributed to early adverse experiences may be more predictive of subsequent psychopathology than the experiences themselves. In evaluating early traumatic or adverse events, clinicians need to evaluate and understand how the patient interpreted those events.
- A full understanding of pathogenic beliefs requires the clinician to evaluate both the occurrence of these beliefs and the distress associated with them, thereby allowing for the assessment of reactivity. The relationship between early adverse events and psychopathology tends to be stronger in individuals with high pathogenic belief reactivity, and these individuals may be more likely to benefit from interventions that reduce the distress associated with them.
- Assessing both the occurrence and distress associated with pathogenic beliefs can potentially provide more precise treatment outcome measurement. At the beginning of therapy, a patient who reported the occurrence of particular pathogenic beliefs and a high degree of distress associated with them may at the end of therapy still report being aware of the beliefs (occurrence) but feel little if any distress connected with them.

*The mind is its own place, and in itself can make a Heav'n of Hell, a Hell of Heav'n*.Milton, Paradise Lost

*All that we are is the result of our thoughts; it is founded on our thoughts and made up of our thoughts. With our thoughts we make the world*.Dhammapada

## 1 Introduction

Stan and Larry, twin brothers, were raised by alcoholic parents who were frequently overwhelmed, neglectful, resentful and often verbally abusive. Stan responded to this adverse parenting by removing himself from the family whenever possible; he actively engaged in team sports and other extracurricular activities, and when he had to be at home, he immersed himself in reading science fiction. Larry, by contrast, internalized his parents' critical, negative comments. They incessantly accused both boys of being selfish and oblivious to pitching in to ‘help the family’. Larry took these criticisms to heart, believed that they were right, and from an early age was determined to improve himself by being a model son. Despite his best efforts, the criticism continued unabated; there was simply nothing he could do to address the bitter resentment his parents chronically felt.

Both brothers did very well academically and went on to successful careers—Stan as a surgeon and Larry as a chief financial officer at a small company. Instead of expressing an appropriate sense of pride in their sons' accomplishments, the parents indignantly conveyed that the boys were now obligated to help support them because they had done so little to ‘help the family’ while growing up. Stan periodically sent small amounts of money and seemed to be relatively immune to the parents' complaints. Larry continued trying to be the model son, supporting them more generously and regularly. He recurrently felt that no matter how much he did, it was never enough and he could never ‘make them happy’. These frustrating feelings led him to seek psychotherapy, and the therapist identified a cluster of beliefs at the root of Larry's suffering: Larry believed that he was responsible for his parents' unhappiness and that he did not do enough to help them, leading to pervasive feelings of (unconscious) guilt.

### 1.1 Pathogenic Beliefs and the Pathogenic Belief Scale (PBS‐21)

Many models of psychopathology and psychotherapy argue that such beliefs play a central role in human suffering and psychological disorders. Cognitive behaviour theorists, for example, refer to core dysfunctional beliefs (Beck 1967, 2011; Beck et al. 1979), rational emotive theorists to irrational beliefs (Ellis 2010) and schema theorists to maladaptive childhood schemas (Young 1990; see also, Beck 2011). Control‐mastery theory, an integrative cognitive‐psychodynamic‐relational framework (Silberschatz 2005; Weiss 1993) refers to pathogenic beliefs—those that impede the pursuit of adaptive goals—typically arising from adverse childhood experiences. Goals emphasized by CMT are typically associated with aspects of adaptive psychological development, such as the development of healthy self‐esteem and self‐direction, capacity for close relationships and pursuit of meaningful work and generativity. Clinically, patients' goals are individualized according to their psychosocial developmental context. Beliefs are pathogenic according to the extent to which they interfere with such goals. Research has shown that these goal obstructing pathogenic beliefs can be reliably identified in psychotherapy sessions (Curtis and Silberschatz 2022; Curtis et al. 1994; Curtis et al. 1988), despite often functioning at an implicit or unconscious level.

While several measures of irrational or dysfunctional beliefs have been developed, their reliabilities are generally poor (Bridges and Harnish 2010; Terjesen et al. 2009). Moreover, all of these measures are based on various theoretical models and assessed the preferred constructs of their respective models—in other words, they were ‘top down’ measures. Developed as a ‘bottom‐up’ scale, the Pathogenic Beliefs Scale (PBS) draws directly from pathogenic beliefs that patients expressed in psychotherapy sessions, thus reflecting beliefs naturally endorsed by patients (Silberschatz and Aafjes‐van Doorn 2017).

The initial version of the PBS (the PBS‐59) was developed and tested on a community sample of 732 adults (Silberschatz and Aafjes‐van Doorn 2017). In contrast to ‘top down’ measures that have previously been developed, the PBS scale consisted of 59 items drawn from psychotherapy sessions. Good reliability and validity data were reported. Subsequent research focused on improving the scale's psychometric properties and replicating results with new community as well as clinical samples (Aafjes‐van Doorn et al. 2021, 2021). The scale has been adapted and tested on a sample of patients across countries in Thailand (Neelapaijit et al. 2017, 2018) and Turkey (Türkkan et al. 2025) with very good results. The most recent iteration of the scale, the PBS‐21 (McCollum et al. 2024) used item‐response theory and confirmatory factor analysis to develop a psychometrically sound 21 item version of the pathogenic belief scale. Three factors have been identified and confirmed across all of the PBS versions—‘undeserving’, ‘can't rely on others’, ‘interpersonal guilt’—with excellent psychometric properties (Aafjes‐van Doorn et al. 2021; McCollum et al. 2024; Türkkan et al. 2025). Examples of PBS‐21 items that are readily applicable to the case of Larry we mentioned previously include ‘I believe I have been unable to make my parents or significant others happy’, ‘I believe I am responsible for the feelings or behaviors of others’, ‘I believe pursuing my own interests and goals mean I am selfish or uncaring’.

### 1.2 Distinguishing the Occurrence, Distress and Reactivity of Pathogenic Beliefs

It is unlikely that Larry was alone in developing pathogenic feelings of responsibility and guilt for his parents' misery. Children who grow up with blaming, abusive parents frequently feel that they, rather than their parents, are at fault (Tanzer et al. 2020). It is therefore likely that both brothers developed pathogenic beliefs of responsibility and guilt toward their parents. One way that the brothers differed was in how reactive they each were to these pathogenic beliefs. Reactivity to pathogenic beliefs means these beliefs may bring a lot of distress and dictate a person's behaviour, attitudes and self‐perception, unchecked by reflection or mentalization. Larry was highly reactive to the belief that he should (or could) do more to make his parents happy and went to great lengths to try to do so. Although he became more aware of these beliefs during the course of psychotherapy, it was the degree of reactivity to the beliefs that changed.

Theoretical and empirical findings suggest a key distinction between the mere existence of pathogenic beliefs, the distress they cause and one's reactivity to them—or how easily that distress is triggered. Recent research on psychological trauma (e.g., Boals 2018) proposes that what is crucial is not just the endorsement of pathogenic beliefs, but also their subjective meaning and impact. In other words, someone may be aware of their pathogenic beliefs but feel less bothered by them, which indicates a low level of reactivity. Cognitive theories propose that *cognitive reactivity*—the activation of a negative schema, rather than its mere existence—is a key pathway to developing depression (Scher et al. 2005). Consequently, assessing both the existence of pathogenic beliefs and an individual's reactivity to them may be crucial for understanding the development and maintenance of psychopathology.

Assessment of reactivity has typically been done through experimental tasks that prime or induce a negative mood, followed by an assessment of dysfunctional beliefs (Scher et al. 2005; Lethbridge and Allen 2008). In contrast, very few studies have explored the possibility of identifying reactivities to pathogenic beliefs through self‐report questionnaires, which are more convenient and accessible for clinical use. To address this need, the most recent version of the PBS (McCollum et al. 2024) includes a two‐part scoring system to distinguish the mere endorsement or occurrence of the pathogenic beliefs and the distress that individuals experienced from the pathogenetic beliefs. Subjects were first asked whether they have the belief (yes/no) and if yes, how distressing the belief was to them. Reactivity to beliefs can be calculated through the comparisons of distress and occurrence of beliefs. The difference between the presence or occurrence of a belief and the associated distress may help us understand why some individuals develop psychopathology and others do not. In the example of Larry and Stan, both brothers might share some similar beliefs, but the associated distress or reactivity to those beliefs may differ. Thus, by separating the occurrence of pathogenic beliefs from the distress and the reactivity associated with them, the latest version of the PBS (PBS21; McCollum et al. 2024) can then be used by researchers to further examine these distinctions and their associations to important predictors and clinical outcomes.

There were two primary objectives for the present study:

1. Previous PBS research found that the relationship between perceived problematic parenting such as abuse or neglect (adverse childhood experiences) and psychopathology is mediated by pathogenic beliefs—a finding recently substantiated in a large scoping review of dozens of studies (Aafjes‐van Doorn, Kamsteeg, et al. 2020). In the current study, we wanted to assess whether the most recent—and most psychometrically validated—version of the PBS (PBS‐21) mediates the relationship between perceived adverse parenting and measures of psychopathology.
2. We also wanted to understand how reactivity to pathogenic beliefs—the discrepancy between distress and the occurrence of these beliefs—may moderate the link between adverse childhood parenting and psychopathology.

## 2 Method

### 2.1 Participant

We recruited 390 participants from the United Kingdom through the online recruitment platform *Prolific.co*. Prolific is known for reducing common online responding biases and for recruiting participants who are more likely to provide meaningful responses. We screened for adult participants who have had experiences as clients in psychotherapy. The age of participants ranged from 18 to 67 (*M* = 34.36, *SD* = 11.02), with 66% identified as female, 30% identified as male and 4% identified as non‐binary or gender queer. The other demographic variables are presented in Table 1.

**TABLE 1:** Demographic characteristics of participants (*N* = 390).

Variable	*n* (%)
Gender	
Female	257 (66%)
Male	115 (30%)
Nonbinary, gender queer, or other	18 (4%)
Race/ethnicity	
European	322 (83%)
Asian or Asian Indian	24 (6%)
Hispanic/Latinx	2 (0.5%)
Middle Eastern	4 (1%)
African	7 (1.8%)
First nations or indigenous	1 (0.3%)
Other	17 (4.4%)
Education	
Less than high school	5 (1.3%)
High school or equivalent	66 (17%)
Some college or 2‐year diploma	108 (28%)
Undergraduate degree	142 (36%)
Graduate degree	69 (18%)
Currently having a mental health problem	314 (81%)
Have been in psychotherapy	374 (96%)

### 2.2 Measure

#### 2.2.1 The Occurrence, Distress and Reactivity of Pathogenic Beliefs

The Pathogenic Beliefs Scale (PBS; Aafjes‐van Doorn et al. 2021; McCollum et al. 2024; Silberschatz and Aafjes‐van Doorn 2017) was used to assess transdiagnostic internalized beliefs that patients expressed in early psychotherapy sessions. Items for the PBS were derived from idiographic case formulations of 21 psychotherapy cases, which included cognitive‐behavioural, time‐limited psychodynamic therapies and long‐term psychodynamic treatments. The original version of the scale (Silberschatz and Aafjes‐van Doorn 2017) was composed of 59 items, with higher scores indicating more severe pathogenic beliefs. Subsequent studies found a three‐factor structure including ‘undeserving’, ‘can't rely on others’ and ‘interpersonal guilt’—with excellent psychometric properties (Aafjes‐van Doorn et al. 2021; McCollum et al. 2024). The most recent PBS version (McCollum et al. 2024), comprising 21 items, was used in the present study. The short version of PBS has confirmed the three‐dimensional factor structure and identified convergent validity with the Dysfunctional Attitudes Scale and the Cognitive Distortions Scale (Türkkan et al. 2025). The PBS‐21scale demonstrated good internal consistency in the present sample, with an alpha coefficient of 0.85 and 0.88 for PBS occurrence and PBS distress. Because the focus of the present study is to examine the mediator role of PBS and because the subscales are highly correlated with the total scale (r ≈ 0.80), we chose to use the total scale of PBS instead of the three subscales.

The unique rating system of PBS allows researchers to calculate three indices: PBS occurrence, PBS distress and the index called PBS reactivity that measures the relative relationship between occurrence and distress. PBS occurrence refers to how many pathogenic beliefs that each participant endorsed. PBS distress refers to the degree of distress each participant experienced from having the pathogenic beliefs. PBS reactivity refers to the relative distress that each participant experienced compared to the number of pathogenic beliefs endorsed.

Specifically, in the 21‐item PBS, participants were asked (1) whether they have the belief (yes/no) and (2) if yes, how distressing the belief is to them—rated from 0 (*not at all*), to 3 (*highly distressing*)—for each item. PBS occurrence is then calculated as the total number of pathogenic beliefs endorsed by each participant. For each belief item, it is possible for participants to endorse the occurrence of the belief but rate little distress from it. The distress for each pathogenic belief item was calculated as PBS distress = rating of PBS occurrence * (patient rating of distress + 1). The PBS distress score for each item is then summed as the total of the PBS distress endorsed by each participant. Because multiplying the occurrence score by a distress score of 0 would result in 0, without the +1 transformation, a score of 0 would correspond to both not holding a belief and holding a belief with little to no distress.

We also identified an index called PBS reactivity to reflect the relationship between PBS occurrence and PBS distress. PBS reactivity is calculated to indicate the proportion of distress generated from the endorsement of occurrence in pathogenic beliefs. First, we standardized the sample based on PBS occurrence and PBS distress and created z scores on PBS occurrence and PBS distress for each participant. PBS reactivity is then calculated using the equation: *PBS reactivity = Z score of PBS distress − Z score of PBS occurrence*. Thus, a PBS reactivity = 0 indicated that the participant endorsed the average level of distress for the number of pathogenic beliefs endorsed (PBS occurrence). A PBS reactivity score > 0 indicated that the participant endorsed more distress than the typical level for the same level of PBS occurrence. A PBS reactivity score < 0 indicated that participants experienced less distress than typically experienced for the same level of PBS occurrence.

#### 2.2.2 Perceived Adverse Parenting in Childhood

The Measure of Parental Style (MOPS; Parker et al. 1997) is a measure of adults' perceptions of dysfunctional parental behaviours and attitudes during their childhood (before age of 17). It is often used as a measure of the likelihood of exposure to dysfunctional parenting, childhood maltreatment or childhood adversity. The MOPS included behaviours indicating child abuse (e.g., verbally abusive and physically abusive), child neglect (e.g., made me unsafe) and unwanted parenting behaviours (e.g., overprotective of me, left me alone, ignored me and made me feel guilty), which can be categorized into three style subscales including indifference, abuse and overcontrol. The MOPS has a maternal form and a paternal form, each consisting of 15 items scored on a 4‐point Likert scale (0–3), with higher scores indicating more perceived adverse parenting in childhood. In the present study, we averaged the maternal and paternal scores to reflect overall perceived adverse parenting in childhood, as done by several other studies (e.g., Aafjes‐van Doorn et al. 2021). The MOPS scale demonstrated good internal consistency in the present sample, with an alpha coefficient of 0.89 and 0.87 for the maternal form and the paternal form respectively.

#### 2.2.3 Depressive and Anxiety Symptoms

We measured depressive and anxiety symptoms using the Patient Health Questionnaire for Depression and Anxiety (PHQ‐4) (Kroenke et al. 2009). PHQ‐4 is an ultra‐brief self‐report screening measure that reliably and validly assesses anxiety and depression. Participants are required to rate the frequency of four key symptoms for the past 2 weeks from *not at all* (0) to *nearly every day* (3). The total score of the four items were used in the current study. The Cronbach's alpha in the current study is 0.84.

#### 2.2.4 General Psychological Distress

The Kessler Psychological Distress Scale (K6) was used to assess participants' general distress levels (Kessler et al. 2002). K6 has been shown to be a reliable and valid self‐report screening measure to assess nonspecific psychological distress and to identify individuals with serious mental illness. In the current study, participants rated the frequency for 6 symptoms from 0 (*none of the time*) to 4 (*all of the time*) for the past 30 days. Cronbach's alpha for K6 in the current study is 0.86.

#### 2.2.5 Interpersonal Problems

The Inventory of Interpersonal Problems (IIP‐32; Soldz et al. 1995) was used to assess interpersonal problems of participants. The IIP‐32 includes 32 items assessing interpersonal problems from the perspective of the interpersonal circumplex. It contains eight octant scales with four items within each scale, assessing interpersonal behaviours that an individual does too much (e.g., I want to be noticed too much) or finds it hard to do (e.g., it's hard for me to join in on groups). Participants were asked to rate the severity on a 0–4 scale for each item. A total score is generated for each of the eight octant scales, and these octant scale scores are used to calculate composite scores. The eight octant scales are organized in the circumplex by two orthogonal dimensions defined by dominance‐submissiveness (agency) and warmth‐coldness (communion). In the current study, we used the total score of interpersonal problems to represent overall severity of interpersonal distress (Luo et al. 2018).

### 2.3 Data Analytic Strategy

We first conducted descriptive analyses and correlational analyses among study variables. To address our first research question, we conducted mediation analyses to examine whether PBS occurrence and PBS distress would mediate the pathway from adverse childhood parenting to three psychopathology outcomes (each outcome in one separate mediation model): general distress, depressive and anxiety symptoms and severity of interpersonal problems. The predictor is perceived adverse parenting in childhood (MOPS). To address our second research question, PBS reactivity was modelled as the moderator for the mediation model for both the path from the predictor (MOPS) to the mediator (PBS occurrence or PBS distress) and the path from the mediator to the outcome in the indirect effect.

We used IBM SPSS Statistics 29.0 for all analyses. Model 4 of the PROCESS macro was used to examine the mediating effects of pathogenic beliefs. Given the exploratory nature of the moderated mediation model, Model 58 was used to examine the moderated mediation with the moderator of PBS reactivity moderating pathways from both predictors to mediator and from mediator to outcome (Hayes 2022). More parsimonious models were conducted when one or more of the moderations in model 58 was not significant. We used the bootstrapping method that conducted 5000 bootstrap samples with 95% confidence intervals (CIs) to test the significance of indirect effects.

## 3 Results

### 3.1 Preliminary Analysis

Descriptives and correlation results are presented in Table 2. PBS occurrence and PBS distress were all significantly correlated with MOPS and three symptom measures (K6, PHQ‐4 and IIP). PBS occurrence and PBS distress were highly correlated with each other (*r* = 0.96, *p* < 0.001). PBS reactivity was significantly correlated with PBS occurrence (*r* = −0.14, *p* = 0.004) and PBS distress (*r* = 0.14, p = 0.004), suggesting that PBS reactivity is a separate concept from PBS occurrence and PBS distress, capturing something that differentially related to PBS occurrence and PBS distress. Additionally, PBS reactivity was not significantly correlated with MOPS (*r* = 0.05, *p* > 0.05), suggesting that participants' reactivity towards pathogenic beliefs were not directly related to their reported adverse childhood parenting experiences.

**TABLE 2:** Descriptives and Pearson correlations for study variables.

Variables	Mean (SD)	1	2	3	4	5	6	7
1. PBS occurrence	9.87 (4.95)	1	0.96**	−0.14	0.28**	0.53**	0.61**	0.73**
2. PBS distress	28.7 (17.43)		1	0.14**	0.29**	0.57**	0.65**	0.76**
3. PBS reactivity	0 (0.29)			1	0.05	0.15**	0.13*	0.09
4. MOPS	14.4 (11.1)				1	0.13*	0.16**	0.32**
5. PHQ‐4	6.2 (3.16)					1	0.85**	0.49**
6. K6	12 (6.54)						1	0.57**
7. IIP severity	0.50 (0.79)							1

*Note:* PBS = Pathogenic Belief Scale (PBS‐21). PBS occurrence = the total number of pathogenic beliefs endorsed by participants. PBS distress = the total distress from pathogenic beliefs endorsed by participants. PBS reactivity = *Z score of PBS distress − Z score of PBS occurrence*. MOPS = The Measure of Parental Style, assessing adverse childhood parenting. PHQ‐4 = Patient Health Questionnaire for Depression and Anxiety; K6 = The Kessler Psychological Distress Scale; IIP = Inventory of interpersonal circumplex.

* Indicates significance at *p* < 0.05.

** Indicates significance at *p* < 0.01.

### 3.2 Mediation Analysis

PBS occurrence (Tables 3 and 4) fully mediated the impact of adverse parenting on depressive and anxiety symptoms (indirect effect = 0.04, SE = 0.008, 95% CI = 0.03–0.06; direct effect = −0.006, SE = 0.01, 95% CI = −0.03–0.02) and general distress (indirect effect = 0.08, SE = 0.01, 95% CI = 0.05–0.11; direct effect = −0.006, SE = 0.02, 95% CI = −0.04–0.03), and partially mediated the impact on interpersonal problems (indirect effect = 0.02, SE = 0.03, 95% CI = 0.01–0.02; direct effect = 0.009, SE = 0.003, 95% CI = 0.003–0.01). The results are the same for PBS distress: It is a full mediator for depressive and anxiety symptoms and general distress and a partial mediator for interpersonal problems (Tables 3 and 4). These results indicated that the presence of pathogenic beliefs and the distress from these beliefs were key mechanisms by which adverse parenting may contribute to adult psychopathology.

**TABLE 3:** Unstandardized coefficients of mediation analyses.

Variables	B	SE	*t*	*p*	LLCI	ULCI
**Mediator: PBS occurrence; outcome: K6**						
MOPS → PBS occurrence	0.13	0.02	5.77	< 0.001	0.08	0.17
PBS occurrence → K6	6.23	0.04	14.58	< 0.001	0.54	0.71
MOPS → K6	−0.006	0.02	−0.31	0.75	−0.04	0.03
**Mediator: PBS occurrence; outcome: PHQ‐4**						
MOPS → PBS occurrence	0.13	0.02	5.77	< 0.001	0.08	0.17
PBS occurrence → PHQ‐4	0.34	0.03	11.81	< 0.001	0.28	0.39
MOPS → PHQ‐4	−0.006	0.01	−0.5	0.62	−0.03	0.02
**Mediator: PBS occurrence; outcome: IIP severity**						
MOPS → PBS occurrence	0.13	0.02	5.65	< 0.001	0.09	0.18
PBS occurrence → IIP	0.12	0.006	18.27	< 0.001	0.1	0.12
MOPS → IIP	0.009	0.003	3.11	0.002	0.003	0.01
**Mediator: PBS distress; outcome: K6**						
MOPS → PBS distress	0.47	0.77	6.06	< 0.001	0.31	0.62
PBS distress → K6	0.19	0.01	16.2	< 0.001	0.17	0.21
MOPS → K6	−0.02	0.02	−0.84	0.4	−0.05	0.02
**Mediator: PBS distress; outcome: PHQ‐4**						
MOPS → PBS distress	0.46	0.08	6.06	< 0.001	0.31	0.62
PBS distress → PHQ‐4	0.1	0.008	13.29	< 0.001	0.09	0.12
MOPS → PHQ‐4	−0.01	0.01	−1	0.32	−0.04	0.01
**Mediator: PBS distress; outcome: IIP severity**						
MOPS → PBS distress	0.5	0.08	6.03	< 0.001	0.33	0.66
PBS distress → IIP	0.03	0.002	19.86	< 0.001	0.03	0.04
MOPS → IIP	−0.007	0.003	2.61	0.01	0.002	0.012

**TABLE 4:** Bootstrap results for indirect effects for the mediation analyses.

Variables	Effects	SE	LL 95% CI	UL 95% CI
**Mediator: PBS occurrence**				
Direct effects of MOPS on K6	−0.006	0.02	−0.04	0.03
Indirect effect of MOPS on K6 via PBS occurrence	0.08	0.01	0.05	0.11
Direct effects of MOPS on PHQ‐4	−0.006	0.01	−0.03	0.02
Indirect effect of MOPS on PHQ‐4 via PBS occurrence	0.04	0.008	0.03	0.06
Direct effects of MOPS on IIP	0.009	0.003	0.003	0.01
Indirect effect of MOPS on IIP via PBS occurrence			0.01	0.02
**Mediator: PBS distress**				
Direct effects of MOPS on K6	−0.02	0.02	−0.05	0.02
Indirect effect of MOPS on K6 via PBS distress	0.09	0.17	0.06	0.12
Direct effects of MOPS on PHQ‐4	−0.01	0.01	−0.04	0.01
Indirect effect of MOPS on PHQ‐4 via PBS distress	0.05	0.009	0.03	0.07
Direct effects of MOPS on IIP	0.007	0.0027	0.002	0.012
Indirect effect of MOPS on IIP via PBS distress	0.02	0.003	0.01	0.02

### 3.3 Moderated Mediation Analysis

**FIGURE 1:**
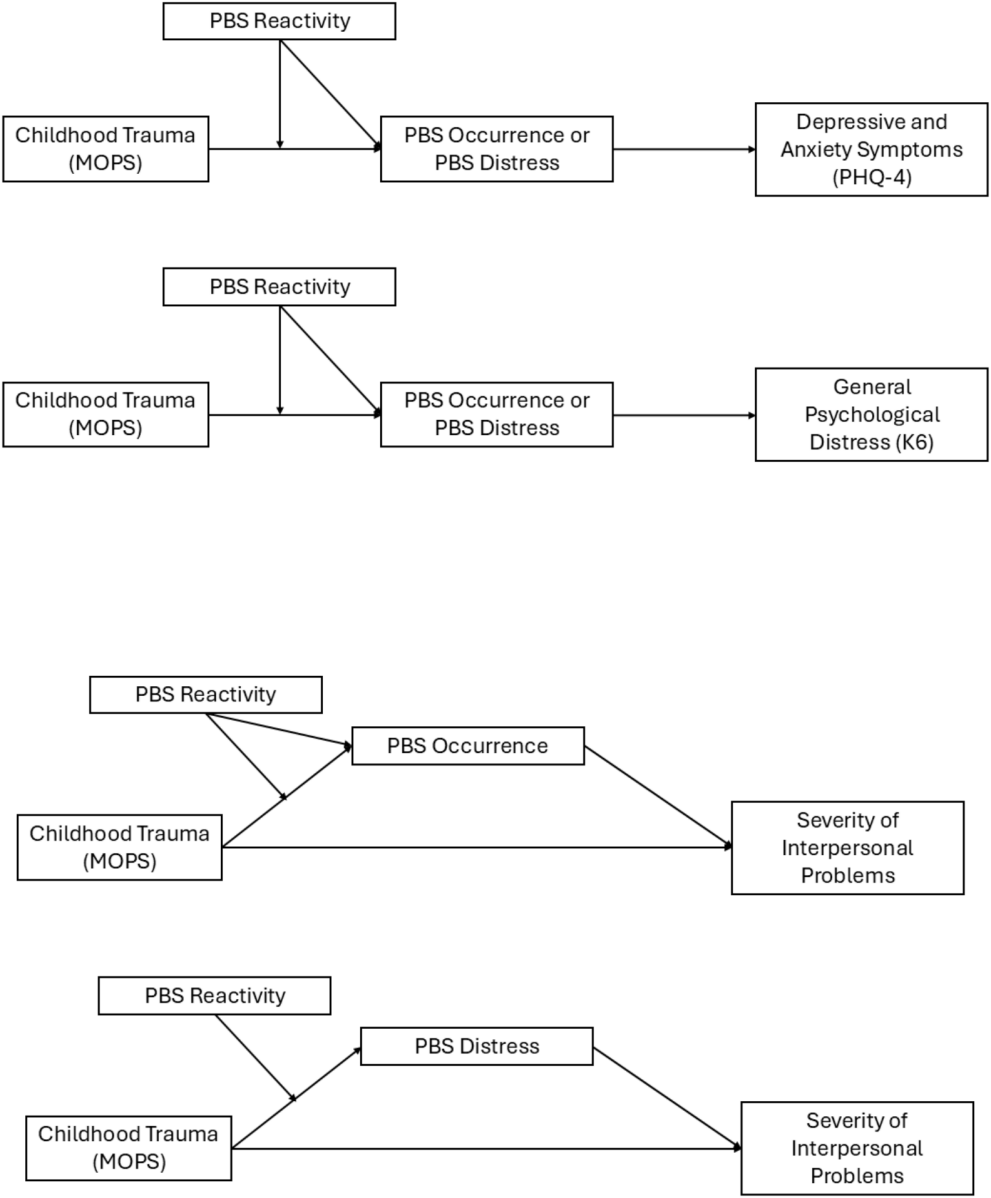
The moderated mediation model (full mediation for K6 and PHQ‐4, and partial mediation for IIP).

We next tested whether PBS reactivity moderated the pathways in the mediation model (Figure 1). The initial model examined moderation at both stages—from MOPS to PBS (path a) and from PBS to symptom outcomes (path b). We found that PBS reactivity significantly moderated the pathway between the predictor (MOPS) and the mediator (PBS occurrence/distress; path a) but did not moderate the pathway between the mediator (PBS occurrence/distress) and the outcomes (path b). Thus, we conducted a more parsimonious model (Model 7) to only include the moderation of PBS reactivity for the pathway between the predictor (MOS) and the mediator (PBS occurrence/distress). The index of moderated mediation was significant across models with all three outcomes (K6, PHQ‐4, IIP) and two mediators (PBS occurrence, PBS distress) (Tables 5, 6, 7).

**TABLE 5:** Moderated mediation analyses with PBS reactivity as the moderator.

Variables	B	SE	*t*	*p*
**Mediator: PBS occurrence**				
Outcome: K6				
MOPS → PBS occurrence	0.12	0.02	5.45	< 0.001
PBS reactivity → PBS occurrence	−3.19	0.82	−3.9	< 0.001
MOPS * PBS reactivity → PBS occurrence	0.29	0.07	4.25	< 0.001
PBS occurrence → K6	0.62	0.04	14.58	< 0.001
MOPS → K6	−0.006	0.02	−0.31	0.75
Outcome: PHQ‐4				
MOPS → PBS occurrence	0.12	0.02	5.45	< 0.001
PBS reactivity → PBS occurrence	−3.19	0.82	−3.9	< 0.001
MOPS * PBS reactivity → PBS occurrence	0.28	0.07	4.25	< 0.001
PBS occurrence → PHQ‐4	0.34	0.03	11.81	< 0.001
MOPS → PHQ‐4	−0.006	0.01	−0.5	0.62
Outcome: IIP severity				
MOPS → PBS occurrence	0.12	0.02	5.42	< 0.001
PBS reactivity → PBS occurrence	−3.48	0.86	−4.03	< 0.001
MOPS * PBS reactivity → PBS occurrence	0.3	0.07	4.24	< 0.001
PBS occurrence → IIP	0.11	0.006	18.27	< 0.001
MOPS → IIP	0.009	0.003	3.11	0.002
**Mediator: PBS distress**				
Outcome: K6				
MOPS → PBS distress	0.41	0.08	5.45	< 0.001
PBS reactivity → PBS distress	6.22	2.88	2.15	0.03
MOPS * PBS reactivity → PBS distress	1.01	0.24	4.25	< 0.001
PBS distress → K6	0.19	0.01	16.2	< 0.001
MOPS → K6	−0.02	0.02	−0.84	0.4
Outcome: PHQ‐4				
MOPS → PBS distress	0.41	0.08	5.45	< 0.001
PBS reactivity → PBS distress	6.22	2.88	2.16	0.03
MOPS * PBS reactivity → PBS distress	1.01	0.24	4.25	< 0.001
PBS distress → PHQ‐4	0.1	0.008	13.29	< 0.001
MOPS → PHQ‐4	−0.01	0.01	−1	0.32
Outcome: IIP severity				
MOPS → PBS distress	0.44	0.08	5.42	< 0.001
PBS reactivity → PBS distress	5.2	3.05	1.71	0.09
MOPS * PBS reactivity → PBS distress	1.06	0.25	4.24	< 0.001
PBS distress → IIP	0.03	0.002	19.86	< 0.001
MOPS → IIP	0.007	0.003	2.61	0.01

**TABLE 6:** Bootstrap results for conditional indirect effects with PBS reactivity as moderator.

Levels of moderator	Effects	BootSE	BootLL 95% CI	BootUL 95% CI
**Mediator: PBS occurrence**				
Direct effects of MOPS on K6	−0.006	0.02	−0.04	0.03
Indirect effects of MOPS → PBS occurrence → K6				
PBS reactivity = −0.28*	0.02	0.02	−0.01	0.06
PBS reactivity = 0.02	0.08	0.01	0.05	0.1
PBS reactivity = 0.27	0.12	0.02	0.08	0.16
Direct effects of MOPS on PHQ‐4	−0.006	0.02	−0.04	0.03
Indirect effects of MOPS → PBS occurrence → PHQ‐4				
PBS reactivity = −0.28*	0.4	0.03	−0.02	0.1
PBS reactivity = 0.02	0.12	0.02	0.08	0.16
PBS reactivity = 0.27	0.19	0.03	0.14	0.25
Direct effects of MOPS on IIP	0.009	0.003	0.003	0.01
Indirect effects of MOPS → PBS occurrence → IIP				
PBS reactivity = −0.30*	0.004	0.003	−0.002	0.01
PBS reactivity = 0.02	0.01	0.003	0.01	0.02
PBS reactivity = 0.27	0.02	0.004	0.02	0.03
**Mediator: PBS distress**				
Direct effects of MOPS on K6	−0.02	0.02	−0.05	0.02
Indirect effects of MOPS → PBS distress → K6				
PBS reactivity = −0.28*	0.02	0.02	−0.01	0.06
PBS reactivity = 0.02	0.08	0.01	0.05	0.11
PBS reactivity = 0.27	0.13	0.02	0.09	0.17
Direct effects of MOPS on PHQ‐4	−0.01	0.01	−0.04	0.01
Indirect effects of MOPS → PBS distress → PHQ‐4				
PBS reactivity = −0.28*	0.01	0.01	−0.006	0.04
PBS reactivity = 0.02	0.05	0.008	0.03	0.06
PBS reactivity = 0.27	0.07	0.01	0.05	0.1
Direct effects of MOPS on IIP	0.007	0.003	0.002	0.012
Indirect effects of MOPS → PBS distress → IIP				
PBS reactivity = −0.30*	0.004	0.003	−0.003	0.01
PBS reactivity = 0.02	0.015	0.003	0.01	0.02
PBS reactivity = 0.27	0.024	0.004	0.02	0.03

* Indicates that when PBS reactivity is lower than −0.28 SD (or −0.30 in some cases), the mediation effects of PBS are no longer significant.

**TABLE 7:** The index and confidence intervals of the moderated mediation.

Moderator: PBS reactivity	Index	BootSE	BootLLCI	BootULCI
MOPS → PBS occurrence → K6	0.18	0.05	0.09	0.27
MOPS → PBS occurrence → PHQ‐4	0.10	0.03	0.05	0.15
MOPS → PBS occurrence → IIP	0.03	0.01	0.02	0.05
MOPS → PBS distress → K6	0.19	0.05	0.10	0.29
MOPS → PBS distress → PHQ‐4	0.11	0.03	0.05	0.16
MOPS → PBS distress → IIP	0.04	0.01	0.02	0.05

**FIGURE 2:**
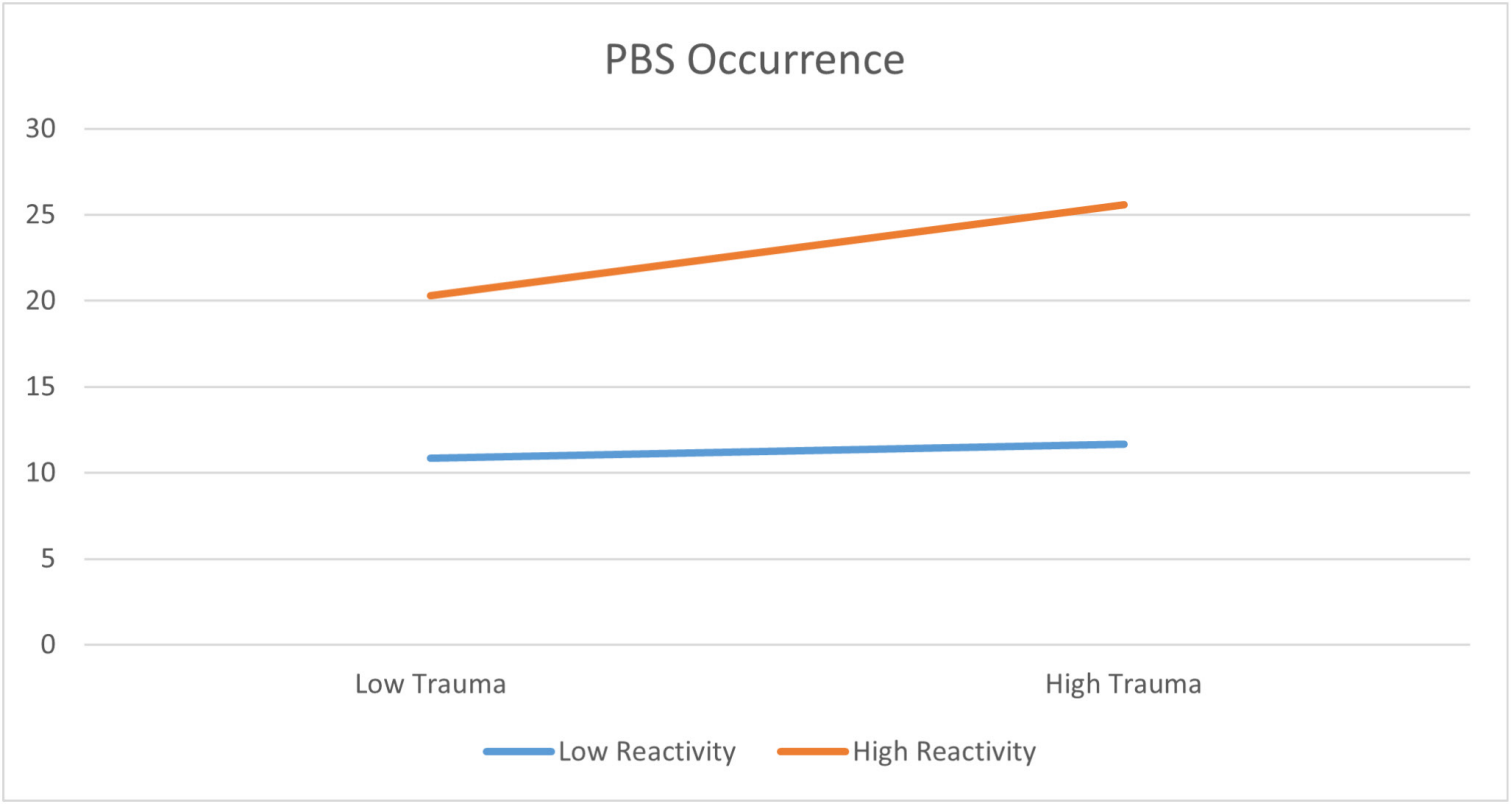
The moderating role of PBS reactivity on PBS occurrence and symptoms (PHQ‐4 as example). *Note:* The high/low levels of PBS reactivity and trauma are defined as ± 1SD from the mean for the scores on PBS reactivity and MOPS, respectively.

These results indicated that when participants had a relatively higher degree of reactivity (*Z* score of reactivity ≥ −0.28), PBS mediates the pathway from adverse parenting to psychopathology outcomes. When participants had a relatively lower degree of reactivity (*Z* score of reactivity < −0.28), PBS does not mediate the pathway and they may internalize less from their adverse parenting in childhood: they reported fewer pathogenic beliefs and experienced less belief‐related distress from childhood trauma (Figure 2).

## 4 Discussion

The results of these analyses are consistent with previous research that demonstrated that pathogenic beliefs mediate the relationship between adverse experiences early in life and later life psychopathology. Furthermore, the relationship between early adverse experiences and psychopathology was moderated by the degree of reactivity observed in participants. For individuals with greater reactivity to pathogenic beliefs (i.e., high distress relative to the level of PBS occurrence), adverse childhood experience is more strongly associated with development of pathogenic beliefs and psychopathology. Consistent with previous studies of pathogenic beliefs (e.g., Aafjes‐van Doorn, Kamsteeg, et al. 2020; Aafjes‐van Doorn, Luo, et al. 2022) and psychological trauma (Boals 2018), there is evidence that adverse experiences themselves may matter less than the meaning individuals make when it comes to the formation of later life psychopathology.

There has been extensive research on the identification of pathogenic beliefs in psychotherapy patients (see Curtis and Silberschatz 2022 for review) as well as empirical studies showing that therapist interventions that disconfirm these beliefs contribute to productive therapeutic processes and effective outcomes (Gazzillo et al. 2022; Silberschatz 2017; Silberschatz and Curtis 1993). These psychotherapy studies have all relied on ratings of pathogenic beliefs by clinical judges. There are also a large number of empirical studies focused on self‐report ratings of pathogenic beliefs (Aafjes‐van Doorn, McCollum, Silberschatz, and Snyder 2021; Gazzillo, Gorman, et al. 2018; McCollum et al. 2024; O'Connor et al. 1997; Sammet et al. 2007). Taken together, these studies have reported consistently good reliability and validity data. For example, significant correlations have been found between interpersonal guilt (a particular type of pathogenic belief) and depression (O'Connor et al. 2002), fear of attachment loss (Gazzillo, Gorman, et al. 2018), l PTSD (McCue et al. 2024) and perfectionism, dependency, and cognitive distortion (Türkkana et al. 2025). These studies together with a recent scoping review (Aafjes‐van Doorn, Kamsteeg, et al. 2020) suggest that cognitions (i.e., pathogenic beliefs) play an important role in the development and maintenance of psychopathology.

Most psychological theories and many neuroscientific models share the assumption that a person's subjective experience or interpretation of events plays a decisive role in emotional adjustment and psychopathology (e.g., Aafjes‐van Doorn, McCollum, Silberschatz, Kealy, and Snyder 2021; Beck 2011; Jin et al. 2023; Luyten and Blatt 2013; Weiss 1993; Young 1990). A recent systematic literature review of 98 studies (comprising over 4000 clinical and 28,000 nonclinical subjects) found that cognitive factors mediate the relationship between adverse childhood experiences and adult psychopathology: ‘it is most likely the internalization of a negative experience in the form of pathogenic cognitive processes that allows negative childhood experiences to have far‐reaching negative consequences’ (Aafjes‐van Doorn, Kamsteeg, et al. 2020, 9).

The current study along with the recent McCollum et al. (2024) research represents a significant advance in the assessment of pathogenic beliefs. First it provides strong psychometric support for the PBS21 and second it illustrates the value of the three‐part scoring system. With the exception of the McCollum et al. study, previous research assessed pathogenic beliefs solely on the basis of occurrence (‘rate the degree to which each belief is applicable or accurate for you’). The assessment of both occurrence and distress allowed for the identification of different subgroups of people with pathogenic beliefs. Even though distress is conditional upon occurrence and the two are therefore highly correlated, one might erroneously assume that occurrence and distress are redundant, but this is not the case. The occurrence and stress ratings allowed us to identify different levels of reactivity to pathogenic beliefs. A person with a high reactivity score is far more likely to have symptoms of psychopathology than someone with a low reactivity score. Identifying these different subtypes would not be possible without distinguishing occurrence from distress.

Previous studies, some using older versions of the PBS, have studied the occurrence of pathogenic beliefs in a variety of different samples recruited from the United States, the United Kingdom, Thailand and Turkey (Aafjes‐van Doorn, McCollum, Silberschatz, Kealy, and Snyder 2021; McCollum et al. 2024; Neelapaijit et al., 2018; Türkkan et al. 2025). Although the versions differed, Türkkan et al. (2025) and McCollum et al. (2024) both found evidence for the three‐dimensions of the PBS; Undeserving, Cannot Rely on Others and Interpersonal Guilt. These results suggest that pathogenic beliefs, as measured by the PBS, are a cross‐cultural phenomenon with implications for clinical practice.

Reactivity, introduced here in this study, may be a useful method for categorizing individuals in research or clinical practice. Because the relationship between adverse experiences and psychopathology was more present or stronger with high reactivity, these individuals may be more likely to hold pathogenic beliefs and more likely to benefit from interventions designed to reduce distress associated with them. It may also be useful, in future research, to specifically examine individuals who have a high occurrence of pathogenic beliefs but low distress associated with them, as this may be a way to identify individuals with high psychological resilience or other adaptive characteristics. Questions remain regarding the reasons for differences in reactivity to pathogenic beliefs, though research on the vulnerability or diathesis‐stress model provide some valuable clues (Elwood et al. 2009; Kugler et al. 2016; McKeever and Huff 2003). Temperament, resilience and emotional processing abilities are among potential factors that could contribute to some individuals developing a higher or lower degree of reactivity. Additional social experiences could also exert a buffering effect for some individuals, allowing one to make use of social support to mitigate their reactivity to a given pathogenic belief. For example, an individual with lower levels of trait neuroticism and greater availability of social support could potentially experience a pathogenic belief as holding less authority over their behaviour than someone with temperamental vulnerability who lacked reliable support figures or other protective factors. Further research is needed to investigate individual difference variables implicated in the development and maintenance of reactivity to pathogenic beliefs.

The results described here also suggest that pathogenic beliefs themselves could be a target for therapeutic interventions as they fully mediate the pathway from trauma to psychopathology. These results are consistent with earlier studies (Aafjes‐van Doorn, Kamsteeg, et al. 2020; Aafjes‐van Doorn, Luo, et al. 2022) examining the mediating role of pathogenic beliefs. Taken together the present study as well as previous work suggests that targeting and measuring pathogenic beliefs in psychotherapy could be a more precise method for understanding the effects of interventions. If, for example, an individual reported holding a distressing pathogenic belief at the beginning of therapy, and then after treatment, reported either no longer holding the belief or still holding it, but at a lower level of distress, this may be a method for a more personalized assessment of improved functioning following psychological trauma.

There are some limitations to the findings presented here. First, this study is cross‐sectional. Although there is an assumed temporal ordering to adverse experiences, the formation of pathogenic beliefs, and later life psychopathology, the data were collected at only a single point in time. It would be useful to examine pathogenic beliefs longitudinally to further understand how they may change over time and the implications they may have for further research and clinical practice. A second important limitation to these results is that all measures included here were self‐report. Self‐report measures have a long history in psychopathology and psychotherapy research, and while generally considered valid, nonetheless are not necessarily better or even equivalent to other methods (Cuijpers et al., 2010; Truijens et al. 2023). In a study on depression, Cuijpers et al. (2010) found that patient self‐reported measures of symptoms tended to be more conservative or show less improvement during therapy. Further studies on pathogenic beliefs could focus on other measurement methods, such as observer rated scores, to understand the potential impact self‐report may have on the findings here.

## Ethics Statement

All procedures followed were in accordance with the ethical standards of the responsible committee on human experimentation (institutional and national) and with the Helsinki Declaration of 1975, as revised in 2000. Informed consent was obtained from all individual participants included in the study.

## Conflicts of Interest

The authors declare no conflicts of interest.

## Data Availability

The data that support the findings of this study are available on request from the corresponding author. The data are not publicly available due to privacy or ethical restrictions.
